# A multi-center, double blind randomized controlled trial evaluating flap fixation after mastectomy using sutures or tissue glue versus conventional closure: protocol for the Seroma reduction After Mastectomy (SAM) trial

**DOI:** 10.1186/s12885-018-4740-8

**Published:** 2018-08-17

**Authors:** J. van Bastelaar, R. Granzier, L. M. van Roozendaal, G. Beets, C. D. Dirksen, Y. Vissers

**Affiliations:** 1 Department of surgery, Zuyderland Medical Center, Sittard-Geleen, the Netherlands; 2 Department of Surgery, Netherlands Cancer Institute, Amsterdam, the Netherlands; 3 GROW School for Oncology and Developmental Biology, University of Maastricht, Maastricht, the Netherlands; 4 Department of Clinical Epidemiology and Medical Technology Assessment, Care and Public Health Research Institute (CAPHRI), Maastricht University Medical Center, Maastricht, Netherlands

**Keywords:** Mastectomy, Seroma formation, Seroma aspiration, Flap fixation, Quilting, Tissue glue, Shoulder function, QALY, Cost-effectiveness

## Abstract

**Background:**

Seroma formation is a common complication after mastectomy and is associated with delayed wound healing, infection, skin flap necrosis, patient discomfort and repeated visits to the out patient clinic to deal with seroma and its sequelae. Closing the dead space after mastectomy seems to be key in reducing seroma and its complications. Various methods have been described to reduce the dead space after mastectomy: closed suction drainage, quilting of the skin flaps and application of adhesive tissue glues. The aim of this trial is to compare seroma formation and its sequelae in the various methods of flap fixation.

**Methods:**

This is a multicenter, double-blind, randomized controlled trial in female breast cancer patients undergoing mastectomy, with or without axillary clearance. Exclusion criteria consist of breast conserving therapy, direct breast reconstruction and incapacity to comprehend implications and extent of study and unable to sign for informed consent. A total of 336 patients will be randomized. Patients will be randomly allocated to one of three treatment arms consisting of flap fixation using ARTISS tissue glue with a low suction drain, flap fixation using sutures and a low suction drain or conventional wound closure (without flap fixation) and low suction drainage. Follow up will be conducted up to twelve months post surgery. The primary outcome is the number of seroma aspirations and secondary outcomes consist of number of out patient clinic visits, surgical skin infection rate, shoulder function, cosmesis, health-related quality of life and costs and cost-effectiveness (cost/QALY).

**Discussion:**

This is the first study of its kind to evaluate the effect of flap fixation and its sequelae (ie seroma aspirations, number of out patient clinic visits, infection, shoulder function, patient assessed cosmesis, quality of life and cost-effectiveness) in a double blind randomized controlled trial.

**Trial registration:**

This trial was approved by the hospitals’ joint medical ethical committee (**14-T-21,** 2 June 2014). The SAM Trial is registered in ClinicalTrials.gov since October 2017, Identifier: NCT03305757.

## Background

Seroma formation is a common side effect after surgery for breast cancer, with a highly variable cited incidence of 3% to more than 90% [1–3]. Seroma is a collection of serous fluid that contains blood plasma and/or lymph fluid. Seroma formation and its sequelae form the mainstay of complications in breast cancer surgery and some surgeons regard it as an unavoidable nuisance after breast cancer surgery. Complications vary from delayed wound healing, repeated seroma aspirations with the risk of infection, prolonged hospital stay, skin flap necrosis, patient discomfort, repeated visits to the out patient clinic, delay in commencing adjuvant therapies and higher surgical expenditures [2, 4, 5].

Many questions still remain with regard to the pathophysiology of seroma formation. Several factors have been held accountable for seroma formation, such as the use of electrocautery, extensive dissection in breast surgery and the extent of axillary lymph node involvement [6–9]. In the last decade, many publications have surfaced, focusing on the surgical prevention of seroma formation following mastectomy and/or axillary clearance. The success of all these interventions seems to have common ground: reduction of the dead space [10]. Closing the dead space after mastectomy can be achieved by closed suction drainage, quilting of the skin flaps or application of adhesive tissue glues to the skin flaps before wound closure [11–15]. There is however no consensus on which technique is most superior in reducing seroma formation and its sequelae [15]. In a randomized controlled trial published in 2010, the authors concluded that it was difficult to elucidate whether reducing the dead space or ligation of lymphatics or a combination of both were responsible for the reduction of seroma formation [16].

The electronic scalpel has been proven to enhance seroma formation after mastectomy [9]. No superior effect in seroma reduction has been seen in the use of other surgical devices (laser scalpel, argon diathermy and ultrasonic scalpel). Van Bemmel et al. concluded that seroma formation after axillary clearance cannot be avoided, but mechanical closure of the dead space leads to a significant reduction of seroma [10]. Several retrospective studies have shown that diminishing the dead space by means of flap anchoring can be very beneficial [17–20].

Until now one prospective randomized controlled trial has been published showing a significant reduction of seroma formation and seroma related complications after flap fixation using quilting sutures [21]. A review published by Van Bastelaar et al. concluded that mechanical flap fixation seems to reduce seroma formation and seroma aspiration after mastectomy with or without axillary clearance. Well executed randomized controlled trials are however needed to confirm these results [22]. The aim of the current randomized controlled trial (RCT) is to compare seroma formation and its sequelae in the various methods of flap fixation. The SPIRIT Statement guidelines were used for designing and describing this trial [23, 24].

## Study objectives

The primary objective of this RCT is to assess the effect of flap fixation using sutures or tissue glue on the number of seroma aspirations after mastectomy for breast cancer in the first year following surgery. Secondary objectives include assessment of the number of out patient clinic visits, infection rate, shoulder function, cosmesis, health-related quality of life, costs and cost-effectiveness.

## Methods

### Study design

The SAM (Seroma reduction After Mastectomy) Trial is a double blind randomized controlled trial. Patients will be allocated to one of three groups. The first group will undergo flap fixation using sutures and placement of low vacuum drainage, the second group will undergo flap fixation using ARTISS tissue glue and low vacuum drain placement and the third group will undergo conventional wound closure and low vacuum drainage.

### Setting

This multi center randomized controlled trial is ongoing at the time of publication and is being conducted at three different district hospitals (Zuyderland Medical Center Sittard, Albert Schweitzer Hospital Dordrecht and St Jansgasthuis Hospital, Weert), two of which are teaching hospitals (Zuyderland Medical Center Sittard and Albert Schweitzer Hospital Dordrecht). All hospitals are situated in the Netherlands.

## Participants

All patients will be recruited from the surgical breast cancer clinics after evaluation for invasive breast cancer or DCIS. Patients will be recruited in three breast cancer clinics, who together treat 1000 patients annually for breast cancer.

### Inclusion criteria

The inclusion criteria are as follows: (1) patients suffering from invasive breast cancer or DCIS with an indication for mastectomy with or without sentinel lymph node biopsy or modified radical mastectomy, (2) female sex, (3) older than 18 years of age.

### Exclusion criteria

The following exclusion criteria will be applied: (1) patients undergoing breast conserving therapy, (2) patients undergoing direct breast reconstruction, (3) patients unable to comprehend implications and extent of study and therefore unable to sign for informed consent.

### Recruitment

The study commenced in June 2014 and the first patient was enrolled on June 14th 2014. The study is still ongoing at the time of publication. Once patients have been screened with respect to the inclusion and exclusion criteria, they are informed about the trial by one of the breast surgeons. After informed consent has been obtained, baseline demographics are noted.

### Randomization

Randomisation is achieved using a web based randomization programme (ALEA, Software for Randomisation in Clinical Trials). Randomization will take place on the day of surgery, after the start of the operation and 30 min before wound closure. Blocked randomization will take place with randomly selected block sizes (6/9/12) with an allocation ratio of 1:1:1. Randomisation will be stratified per site. Both patients and surgeons will be blinded. Patients will be blinded throughout the trial and the surgeon performing the follow-up assessments is also blinded. To ensure blinding, patients will not be evaluated by their own surgeon during follow up. Patients will be allocated into one of three groups, (A) no flap fixation and placement of a low suction drain, (B) flap fixation using sutures and placement of a low suction drain, (C) flap fixation using tissue glue (ARTISS) and placement of a low suction drain.

### Study interventions

All procedures are performed by experienced breast surgeons. The nipple-areola complex is removed and dissection of the skin flaps is performed by using electrocautery. Removal of breast tissue from the pectoral muscle includes removal of the pre-pectoral fascia. All patients receive a low suction drain before closure of the skin. All procedures are performed as day cases unless comorbidities warrant hospital admission.

#### Conventional wound closure

After mastectomy and placement of a low suction drain, extent of the skin flaps will be measured and noted in the CRF. The skin edges will be sutured in one layer using absorbable monofilament sutures (Monocryl 3.0 or V-lok 30 cm), depending on the surgeon’s preference.

#### Flap fixation using sutures

After having performed the mastectomy, extent of the skin flaps is measured (in cm’s) from medial to lateral and from cranial to caudal. The skin flaps will be sutured on to the pectoral muscle using polyfilament absorbable sutures (Vicryl 3.0), placed at 4–5 cm intervals in two or three rows, depending on the extent of the skin flaps. The distance between all sutures is 4–5 cm. Care will be taken to prevent dimpling of the skin. The number of rows and total number of Vicryl sutures are noted in the CRF. The axillary area is not approximated using sutures. The skin edges will be sutured in one layer using absorbable monofilament sutures (Monocryl 3.0 or V-lok 30 cm), depending on the surgeon’s preference.

#### Flap fixation using ARTISS tissue glue

After having performed the mastectomy, extent of the skin flaps is measured (in cm’s) from medial to lateral and from cranial to caudal. ARTISS is applied as a 2 mL spray and used on both skin flaps. Care will be taken to make sure that the skin flap and pectoral muscle surfaces are dry before applying the glue. After the spray has been applied, compression on both skin flaps to the underlying muscle is applied for 3 min. The skin edges will be sutured in one layer using absorbable monofilament sutures (Monocryl 3.0 or V-lok 30 cm), depending on the surgeon’s preference.

All surgeons receive detailed live and video instructions on performing the various closure techniques to standardize closure techniques.

#### Drains

The drain is connected to a low suction drain bottle (Armstrongmedical) and drain output is noted daily. In patients undergoing mastectomy without axillary clearance, the drain is removed when drain output is less than 50 mL or after a maximum of 48 h, irrespective of drain output.. In patients undergoing modified radical mastectomy (including axillary clearance) the drain is removed if daily production is less than 50 mL or after a maximum of 5 days, irrespective of drain output. Patients never receive more than one drain.

##### Interleukin-6 and TNF-α sampling

Pro-inflammatory cytokines such as IL-6 and TNF- α are related to tissue damage. It is expected that patients with higher levels of the early systemic inflammatory response markers in seroma might suffer from increased seroma formation. The aim of this sampling was to assess if there was any association between IL-6 and TNF-α levels in seroma fluid measured on the first postoperative day and seroma and seroma related complications in patients undergoing mastectomy with or without flap fixation. Seroma samples will be collected on the first postoperative day for analysis of Interleukin-6 (IL-6) and Tumor Necrosis Factor (TNF-α). Drain fluid (10 ml) will be collected between 8 and 10 a.m. The samples are centrifuged at 1300 rpm for 10 min and stored at − 80 °C. Human Il-6 and TNF-α concentrations will be determined using in-house developed standard enzyme linked immunosorbent assays (ELISA). The sampling will be performed in one batch to ensure reliability of the sampling process.

##### Il-6 ELISA

96-well microplate (Greiner, 655,061) will be coated overnight at 4 °C with mouse anti- human IL6 (5E1). Hu IL6 (R&D Systems) will be used for standard titration curve. Standard and samples will be incubated for 2 h at room temperature (RT). Biotinylated polyclonal rabbit anti-human IL6 will be bound to captured human IL6. Streptavidin-peroxidase conjugate will be bound to the biotinylated antibody and reacted with the substrate, Tetramethylbenzidine (TMB). The enzyme reaction will be stopped by the addition of 1 M H_2_SO_4_. Spectrophotometry will be performed at 450 nm.

##### TNFα ELISA

96-well microplate (Greiner, 655,061) will be coated overnight at 4 °C with mouse anti- human TNFα (61E71; Celltech). rHu TNFα will be used for standard titration curve. Standard and samples will be incubated for 1 h at room temperature (RT). Polyclonal rabbit anti-human TNFα will be bound to captured human TNFα. Goat anti rabbit peroxidase (IgG HRP Jackson Immuno Research) will be bound to the secondary antibody and reacted with the substrate, Tetramethylbenzidine (TMB). The enzyme reaction will be stopped by the addition of 1 M H_2_SO_4_. Spectrophotometry will be performed at 450 nm.

### Study outcomes

#### Primary outcome

The primary outcome is the number of seroma aspirations, as measured by the number of needle aspirations performed during the first post-operative year. We decided on seroma aspiration as primary outcome, as this probably is the most objective assessment of clinically relevant seroma formation. There are strict criteria for seroma aspiration; the mere presence of seroma does not warrant aspiration. Seroma aspirations will be performed if 1) wound healing is at risk due to seroma (wound break down, seroma leakage, necrosis), 2) if there is discomfort or pain caused by large amounts of seroma, characterised by tenseness of the skin or 3) if there is contaminated/infected seroma and aspiration is necessary to treat infection. All patients that undergo seroma aspiration due to infection will also be treated with a 1 week course of Augmentin 625 mg 3 times daily. All surgeons and nurse practitioners participating in the study have received strict and clear instructions on when to perform seroma aspiration. Adherence to the aforementioned criteria is pivotal for assessing the primary study endpoint. As mentioned previously, all assessors have been blinded to the closure technique used.

#### Secondary outcomes

Secondary outcomes include:

1. The number of out patient clinic visits, measured during the first postoperative year. Patients with complications after mastectomy will have more frequent visits to the breast clinic.

2. Infection rate, as measured by 1) the need for antibiotics, 2) seroma aspiration due to infection or 3) surgical drainage during the first post-operative year.

3. Shoulder function, as measured using the DASH questionnaire (Disability of Arm, Shoulder and Hand, Dutch version). This will be assessed at baseline and at every postoperative visit to the outpatient clinic during one year after surgery. The DASH score has been proven to have a very good reliability and is able to differentiate between shoulder disability levels [6, 7].

4. Cosmesis, as measured using a scale questionnaire, assessed at every postoperative visit to the outpatient clinic during one year after surgery. Patients are questioned on the perceived aspect of their chest wall. Patients will be required to fill in a grading on a Likert scale, from 1 to 10.

5. Health related quality of life using the EQ-5D-5 L, societal costs and cost-effectiveness (cost per Quality Adjusted Life Year; QALY) with a time horizon of 12 months. The EQ-5D will be assessed at baseline, and at postoperative visits at 6 weeks, 3, 6 and 12 months. Costs will be measured with a retrospective cost questionnaire at baseline, and at postoperative visits at 3, 6 and 12 months.

### Follow up

**Fig. 1:**
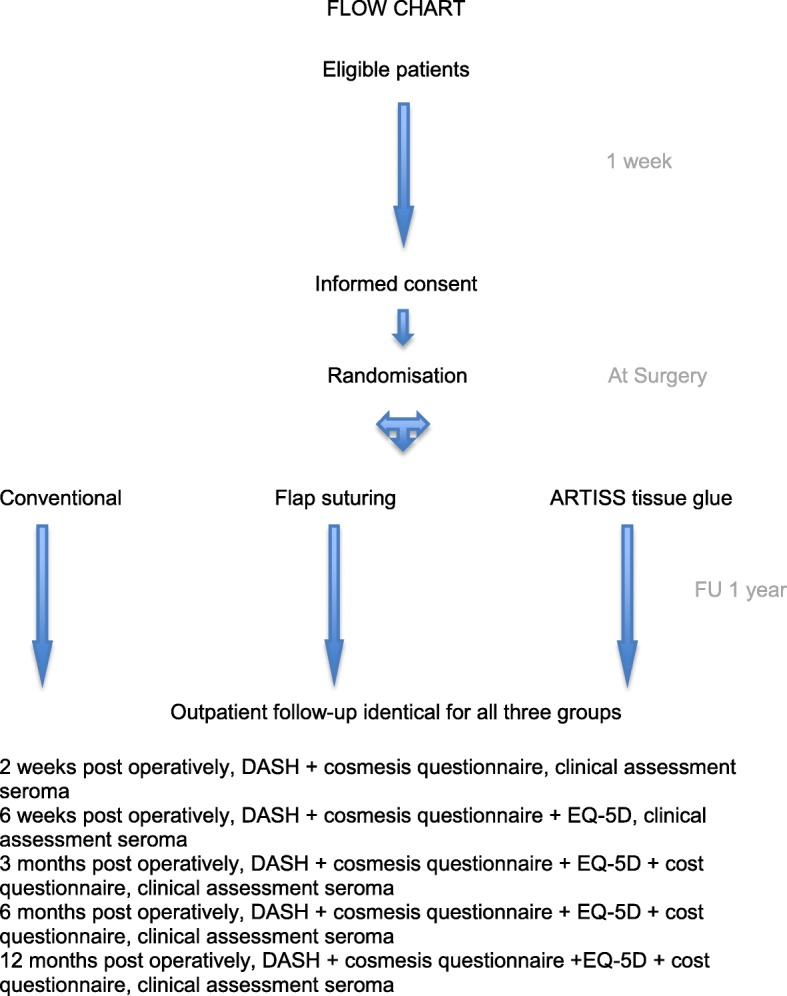
Follow-up schedule

Follow up will be performed until one year after surgery. Patients will be evaluated in the out patient clinic 2 weeks, 6 weeks, 3 months, 6 months and 12 months postoperatively. A figure of the follow-up schedule is visible in Fig. 1. Subjects can leave the study at any time for any reason if they wish to do so without any consequences. The investigator can decide to withdraw a subject from the study for urgent medical reasons. Table 1 represents the time schedule of enrolment, interventions and assessments.

**Table 1:** Time schedule of enrolment, interventions and assessments

TIMEPOINT	Baseline	Day of surgery	Follow up					
	-t_1_	0	Day 1	2 wk	6 wk	3 m	6 m	12 months
ENROLMENT:								
Eligibility screen	X							
Informed consent	X							
*Demographics*	X							
Randomization		X						
INTERVENTIONS:								
Conventional wound closure		X						
Flap fixation using sutures		X						
Flap fixation using tissue glue		X						
ASSESSMENTS:								
Clinical assessment seroma and complications				X	X	X	X	X
Pain score				X	X	X	X	X
Patient reported cosmesis assessment				X	X	X	X	X
DASH	X			X	X	X	X	X
EQ-5D	X				X	X	X	X
Cost evaluation	X		X			X	X	X

## Blinding

Both patients and surgeons will be blinded with regard to if and which method of flap fixation is used. The surgeon performing the procedure is obviously not blinded; surgeons performing the assessments during follow up will be blinded to the closure technique. 30 min before closure patients will be randomized in the online randomization programme ALEA. A randomization number is issued and is duly noted in patients’ files. The operating report will not specify which method has been used. The following statement is noted at the end of the operating report:

‘The trial participant was randomized in 1 of the 3 treatment arms of the SAM Trial. For blinding purposes, this data has been stored in a secured database. In case of SAE or SAR, the method of wound closure can be revealed after contacting the primary investigator.

Postoperatively, patients are not informed of the method of wound closure that was used. Patients and surgeons will be questioned during every postoperative follow up appointment on the presumed method of closure. In this fashion, one will be able to assess whether double blind randomization is realistic upon evaluation. As patients and surgeons are blinded to the method of closure; seroma and its sequelae in trial patients will not be assessed by their own surgeons during follow up.

## Data management

Data is recorded on trial specific case report forms (CRF’s). The following patient demographics and characteristics were recorded: age, BMI, Charlson Comorbidity Index, use of anticoagulants, smoking, neoadjuvant chemotherapy, clinical node positivity, axillary lymph node clearance, TNM staging, specimen weight, mastectomy wound surface, drain output and drain time. To maintain anonymity, CRF’s are identified only by a randomization code. A data manager and research nurse regularly verify data and send queries for missing or inconsistent data. All data is stored in a database that is updated on a weekly basis.

## Sample size

The number of seroma aspirations is not normally distributed. Therefore, sample size estimations based on normally distributed continuous variables cannot be used. It is possible to use formulas based on non-parametric analysis methods, estimating the chance of a random patient in the treatment group having fewer aspirations than a random patient in the control group. However, since the majority of patients will have the same number of aspirations (i.e. 0), this method of sample size estimation does not seem to be applicable to our study. Sample size estimation based on ordinal regression is in line with the data distribution and limited possibilities (maximum number of aspirations in the observational retrospective study was 4) of the outcome variable (Walters SJ. Health Qual Life Outcomes 2004: 2(1); 26). Using alpha = 0.025 (correction for 2 comparisons with 3 study groups), beta = 0.10, and a proportional odds ratio = 2.67 (corresponding to an absolute difference of 20% in the need for seroma aspirations), the sample size is estimated at 112 patients per study group. Therefore a sample size of 336 is planned for. To recruit this sample size, a 40 month inclusion period is estimated.

### Statistical analysis

Missing data will be imputed using stochastic regression imputation to prevent a loss of statistical precision and reduce the likelihood of biased estimates of treatment effect. Mean and standard deviation and absolute number and percentage will describe patient characteristics. Single and multiple ordinal regression analysis will be used to compute the difference in the number of seroma aspirations between the three groups. Secondary outcome parameters will be compared between groups using single and multiple ordinal regression that fits the distribution of that endpoint (e.g. continuous, binary, count). The primary analysis is an intention-to-treat analysis and therefore withdrawals and non-adherence will be analysed in the group to which they were randomized.

### Cost effectiveness

For the economic evaluation, incremental cost-effectiveness ratios will be calculated based on the societal costs per QALY within 12 months. For calculation of the QALY, the EQ-5D-5 L will be assessed at baseline, and at postoperative visits at 6 weeks, 3, 6 and 12 months. Costs will be measured by a retrospective cost questionnaire at baseline, and at postoperative visits at 3, 6 and 12 months. Standard sensitivity analyses and non-parametric bootstrap analyses will be performed to address uncertainty about the cost-effectiveness ratio (s). Cost-effectiveness acceptability curves will be constructed to address the probability that either one of the techniques is cost-effective.

### Monitoring

No data monitoring committee was formed due to the short duration of patient participation and known minimal risks in all arms. Interim analysis is planned after half of the patients have been included. Adverse events will be collected and reported according to guidelines. Yearly updates of trial progress will be reported to the medical ethical committee.

### Ethics and dissemination

In conformity with the Declaration of Helsinki, all participants will be required to sign for informed consent. Informed consent describes the study in detail containing the relevant information enabling patients to make an informed decision about their participation. Consent will be obtained in the breast clinic before patients are scheduled for surgery. Trial participants may withdraw from the study at any time during the trial without their withdrawal impacting further treatment. A formal amendment to the local research ethics committee will be required for any amendments to the study protocol which may impact the conduct of the study. Publications will follow international guidelines: CONSORT Statement. Research findings will be submitted to peer-reviewed journals regardless of whether results are statistically significant.

## Discussion

A number of retrospective trials were performed to demonstrate that flap anchoring and therefore dead space reduction could be very beneficial. Until now one prospective randomized controlled trial has been published showing a significant reduction of seroma formation and seroma related complications after flap fixation using quilting sutures. A review published by Van Bastelaar et al. concluded that mechanical flap fixation seems to reduce seroma formation and seroma aspiration after mastectomy with or without axillary clearance. Well executed randomized controlled trials are however needed to confirm these results and to compare the different flap fixation techniques [22]. Furthermore, no cost-effectiveness analyses have been performed on this topic yet. One other multi center randomized controlled trial (QUISERMAS) is currently being conducted in France to assess the effect of quilting of the dead space after mastectomy on seroma prevention [25]. However patients and surgeons are not blinded to the closure technique used. Moreover patients undergoing mastectomy without axillary clearance in the conventional closure group receive a drain, while the patients in the quilting group are not drained. This may potentially bias the study outcomes and therefore we chose to perform.

a double-blinded study. Furthermore, the QUISERMAS trial does not include a study arm analysing the application of tissue glues. Cosmetic results in the QUISERMAS study will be assessed by an independent adjudication committee. Finally, in our study we decided to let patients assess cosmesis themselves as we consider their opinions as being most important in evaluating outcome. This is the first study of its kind to evaluate the effect of flap fixation and its sequelae (ie seroma aspirations, number of out patient clinic visits, infection, shoulder function, patient assessed cosmesis, quality of life and cost-effectiveness) in a double-blind randomized controlled trial.

## Abbreviations

CONSORT: CONsolidated Standards Of Reporting Trials
CRF: Case report forms
DCIS: Ductal Carcinoma in Situ
EQ-5D: EuroQol-5D quality of life questionnaire
MRM: modified radical mastectomy
QALY: Quality adjusted life year
QUISERMAS: French RCT evaluating QUIlting for the prevention of SERoma after MAStectomy
RCT: Randomized Controlled Trial
SAE: Serious Adverse Event
SAM Trial: Seroma reduction After Mastectomy
SAR: Serious Adverse Reaction
